# Women and the Insurance Bill

**Published:** 1911-07-22

**Authors:** 


### WOMEN AND THE INSURANCE BILL.

The Insurance Bill continues to meet with criticism from every class of women who may be expected to come under its provisions. First, there is the grievance of the married women who, having contributed to the fund during their years of wage-work, find themselves deprived of all benefit from their contributions if, after marriage, they do not go out into the labour market. It is certainly not to the interest of the community that married women should find work outside their homes, but this Bill seems to offer a premium on outside industry. The married woman who falls ill gets 7s. 6d. a week if she is a wage-earner, but nothing if her work is confined to the home. A restriction is put on this apparent advantage by the maternity insurance, which assures a benefit of 30s. to every mother whose husband earns less than £160 a year, whether she goes out to work or not. But unfortunately this fact strikes the women workers as an injustice. If Mrs. Smith, who has the best and most solvent of husbands, gets this 30s. when she has a baby, why should Mrs. Jones, who, owing to the mental, moral, or physical defects of her husband, has to earn her own living and that of the family, not get, in addition to the maternity benefit that comes out of the men's fund—as Mr. Lloyd George assures us!—get the sickness benefit which she has earned by her own work and contributions? It might be urged that—Mr. Jones being what he is—she and her baby are profiting by the contributions of Mr. Smith and his like. But it is hopeless to convince the woman who is going into the labour market to earn her living that she is not paying more than her neighbour and reaping no greater advantage. The logical course would be to drop the maternity benefit altogether—leaving the man in work to provide for these family incidents—and let the insured woman have her sick benefit when laid aside from work, from whatever cause. But if motherhood, and motherhood under good conditions, be a desideratum of the State, it will be necessary to make some arrangement by which, in the case of women who have to earn their own living, the 30s. allowed for confinement expenses—no lavish allowance, it will be admitted!—may be supplemented by a sickness allowance. In the interests of morality we may grant that in the case of unwedded mothers the maternity allowance may be regarded as a sickness allowance—and in most of these cases there will be no responsibilities beyond those of the mother and the new-born child. But in the case of married women thrust on to the labour market long after they had thought that their life was to be passed within the precincts of the home, some wider latitude must be allowed. It is necessary to be just. That is to say: if a woman be the sole bread-winner, let her have, during her absence from work, just the right of the woman bread-winner—her 7s. 6d. a week. But if her husband also is earning—and paying his contribution—she has surely a right to what she earns as well as to what is given to him—that is, her sickness benefit as well as the maternity benefit which is given out of the men's funds. The question is not an easy one, and in matters of politics the line of absolute justice is hard to find; but the claims of woman as mother and wage-earner should be differentiated if the Biill is to be really satisfactory.

Among other women who feel that this National Bill does not meet their needs are the many who "live in." This includes all domestic servants, many workers in the drapery trade, the majority of hotel workers, and hospital nurses. Clause 7 of the Act says that "no insured person shall be entitled (*e*) to sickness or disablement benefit during any period when he is provided with board and lodging by his employer. (This clause will probably fall hard upon medical men, who might reasonably expect to have their just claims for treatment met out of the insurance money, but are defeated by this clause if the patient is during the period of sickness "provided with board and lodging by his employer." For the employer is not compelled to pay wages, from which the cost of medical treatment might be defrayed.) But, as a matter of fact, comparatively few of the people who "live in" are provided with board and lodging during sickness by their employer. In houses where several servants are kept it is, we think, the honourable rule that they should be not only housed and fed, but medically treated at their masters' expense; but where there is only one servant, and only one servant's room, all of this is impossible. And in all the other cases covered by "living in," it is—without any question of harshness or cruelty—frankly out of the question. The draper's assistant, the barmaid, the hotel waiter, who falls ill is sent—and that without any malice, any intention of escaping responsibility, but simply as a result of the necessity of the case—to the nearest hospital or infirmary. The hospital or infirmary gets nothing out of the case, no compensation for the inevitable cost incurred; neither does the employee nor the employer, both of whom have paid their contributions. Either the charitable public or the ratepayers have to bear the cost, while the parties who have contributed to meet the charge feel that, so far as they are concerned, the money has been given for nothing—or at best for the benefit of others. It is not to be expected that such workers will accept with equanimity the idea of making contributions from which they will receive no benefit. Moreover, though the employer is compelled to make insurance against the possible sickness of the worker, there seems to be no provision for relieving him from his responsibility, under the Workmen's Compensation Act, of making up to his employee the wages the latter would otherwise lose through sickness. Thus—to take a hypothetical but not impossible case—typhoid fever might develop among the girls employed in a draper's shop. The conditions from which the disease sprang might be quite beyond his control, for a mere tenant cannot always rearrange the sanitation of his premises to his heart's desire. Yet he would be liable for their wages under the Workmen's Compensation Act if it could be shown that the employees incurred the disease in the discharge of their work. And yet, although both he and the employees had been paying their contributions to the National Insurance Bill, neither he nor the latter would benefit, for the patients would be removed to a hospital, to which the employer might be contributing as a ratepayer! And since, as a taxpayer, the employer would have to give his share to whatever increased taxation might be demanded by the "State" contribution—so many people seem to forget that the so-called State is just the taxpayers taken *en masse*!—he might reasonably complain that he had paid twice over for a benefit from which his employee received nothing; or, alternatively, that having made provision for his employee's needs under the Workmen's Compensation Act, he was compelled, further, to insure them against any sickness, during which they might be treated. Some harmonisation between the National Assurance Bill and the Workmen's Compensation Act is certainly required, and also some arrangement by which assured persons treated either in voluntary hospitals or Poor-law infirmaries should contribute something towards the cost of their treatment.

The position of women who, after having been compelled to contribute through their years of wage-earning, lose all through marrying, has been exploited so much in the Press that there is no need to deal with it here. But the question of a surrender value on such insurance policies is well worth considering. For, after all, the bulk of women who would come under the provisions of the Bill do not return to wage-earning after their marriage; and of the rest who, as widows, have to come back to the labour market, the majority, for the sake of their children, take up such employment as charing and the like, which do not give them the opportunity of re-insuring. What the contributions of the younger women may bring towards the necessities of the older ones is still an unknown quantity; but it seems questionable if in a truly "national" scheme of insurance the interests of the two sexes, so intimately connected, should be actuarially differentiated.

